# Challenges in the control of Human African Trypanosomiasis in the Mpika district of Zambia

**DOI:** 10.1186/1756-0500-6-180

**Published:** 2013-05-04

**Authors:** Victor Mwanakasale, Peter Songolo, Victor Daka

**Affiliations:** 1 Tropical Diseases Research Centre, Ndola, Zambia; 2 World Health Organization, Country Office, Lusaka, Zambia; 3 School of Medicine, Copperbelt University, Ndola, Zambia

**Keywords:** Human African Trypanosomiasis, Elimination, Endemic, Management, Diagnosis, Referral system

## Abstract

**Background:**

Human African Trypanosomiasis is one of the Neglected Tropical Diseases that is targeted for elimination by the World Health Organization. Strong health delivery system in endemic countries is required for a control program to eliminate this disease. In Zambia, Human African Trypanosomiasis is lowly endemic in the northeastern part of the country.

**Findings:**

We conducted a cross-sectional survey of health institutions in Mpika district in Northern Province of Zambia from 9^th^ to 23^rd^ November 2011. The aim of this study was to assess current health delivery system in the management of Human African Trypanosomiasis cases in Mpika district, Northern Province of Zambia. Ten health institutions were covered in the survey. Two structured questionnaires targeting health workers were used to collect the data on general knowledge on HAT and state of health care facilities in relation to HAT management from the surveyed health institution.

Only 46% of the 28 respondents scored more than 50% from the questionnaire on general knowledge about Human African Trypanosomiasis disease. None of the respondents knew how to differentiate the two clinical stages of Human African Trypanosomiasis disease. There were only three medical doctors to attend to all Human African Trypanosomiasis cases and other diseases at the only diagnostic and treatment hospital in Mpika district. The supply of antitrypanosomal drugs to the only treatment centre was erratic. Only one refresher course on Human African Trypanosomiasis case diagnosis and management for health staff in the district had been organized by the Ministry of Health in conjunction with the World Health Organization in the district in 2009. The referral system for suspected Human African Trypanosomiasis cases from Rural Health Centres (RHCs) to the diagnostic/treatment centre was inefficient.

**Conclusions:**

There are a number of challenges that have been identified and need to be addressed if Human African Trypanosomiasis is to be eliminated in a lowly endemic country such as Zambia. These include shortage of trained health workers, inadequate diagnostic and treatment centres, lack of more sensitive laboratory diagnostic techniques, shortage of trypanosomicides among others discussed in detail here.

## Findings

### Background

Human African Trypanosomiasis (HAT), caused by a haemoflagellate of the species *Trypanosoma brucei rhodesiense* in East and Southern Africa, is still endemic at a very low scale in North-eastern Zambia [1]. The districts that have reported sporadic cases of HAT in recent years are Mpika (Northern province, now moved to the newly created Muchinga province), Chama (Eastern province, now moved to the newly created Muchinga province), and Nyimba (Eastern province). Among these districts, Nyimba reported the latest three HAT cases in January 2011 [2].

In Zambia, HAT is transmitted to humans by the bite of an infected tsetse belonging to the species *Glossina morsitans morsitans*, *Glossina morsitans centralis*, *Glossina pallidipes,* and *Glossina brevipalpis*[3]. Over the past four decades the incidence of HAT has been on the decline in Zambia [1]. This decline in incidence of HAT has not been confined to Zambia but to the rest of Africa where this condition has been endemic [4]. HAT is one of the diseases that have been targeted by the World Health organization for elimination [5,6]. In order for any country to eliminate HAT there is need to have a strong health system in place for early identification of clinical signs and symptoms suspicion of HAT, referral system, laboratory diagnosis, and effective treatment instituted early enough to minimize serious drug reactions and mortality [7].

It is for this reason that we conducted a study entitled “Assessment of health delivery system in the management of Human African Trypanosomiasis (HAT) cases in Mpika District”. The aim of this study was to assess current health delivery system in the management of HAT cases in Mpika district, Northern Province of Zambia, Southern Africa. The specific objectives of the study were: To determine levels of knowledge of health staff present in both rural health centres (RHCs) and outpatient departments of the two district hospitals in Mpika District about HAT disease, describe referral system that was in place for HAT cases both at RHCs and districts hospitals in Mpika district, describe diagnostic methods that were available for HAT diagnosis both at RHCs and district hospitals in Mpika district, determine staffing levels that were available to manage HAT cases and antitrypanosomal drug availability at both RHCs and district hospitals, map location of RHCs in areas known to be origins of HAT cases and describe the terrain between these RHCs and the two district hospitals, make recommendations to the Ministry of Health on effective strategies to control HAT in Mpika district.

In this article we describe the methodology, the results obtained, discuss the findings, and make suggestions on how to address the challenges in eliminating HAT in Zambia.

### Methods

#### Study period

The study was conducted from 9th to 23rd November 2011.

#### Study design

The study was a cross-sectional survey of health institutions and involved collection of data using two structured questionnaires. Additional file 1: Questionnaire 1 assessed the general knowledge of health staff on HAT disease (causative agent, transmission, clinical symptoms and signs, treatment, and control) of surveyed health institution. Additional file 1: Questionnaire 2 addressed aspects of management of HAT cases (staffing levels, training of health staff in HAT diagnosis and clinical management, laboratory services in relation to HAT diagnosis, availability of antitrypanosomal drugs, and referral system in place for suspected HAT cases at the surveyed health institution).

#### Study site

The health institutions that were covered in the survey were: Mpika District Hospital, Chilonga Mission Hospital, Nabwalya RHC, Chiundaponde RHC, Muwele RHC, Chalabesa RHC, Kabinga RHC, Mpumba RHC, and Lubunga RHC. Nabwalya RHC was the remotest and hardest to reach RHC. It lies in the historical Luangwa valley [7].

### Field work

On the day of the survey at each surveyed health institution written Informed Consent was obtained from each respondent before answering Additional file 1: Questionnaire 1 and Questionnaire 2. Respondents to the Additional file 1: Questionnaire 1 was all trained health workers in the Outpatient departments of the two hospitals and RHCs on duty on the day of the survey. The respondents answered the questions under the observation of the co-investigator.

Additional file 1: Questionnaire 2 was administered to the respective respondents by the Principal Investigator. Respondents to Additional file 1: Questionnaire 2 was Heads of the surveyed health institutions and selected subordinates present on the day of the survey. The principal Investigator entered the responses to the questions in Additional file 1: Questionnaire 2 at each surveyed institution.

The location of the surveyed health institutions were plotted using the Garmin Global Positioning System (GPS) equipment.

### Ethical consideration

Ethical approval to conduct this study was obtained from the Tropical Diseases Research Centre Ethics committee (Institutional Review Board Number: 00002911).

### Results

#### Maps

**Figure 1:**
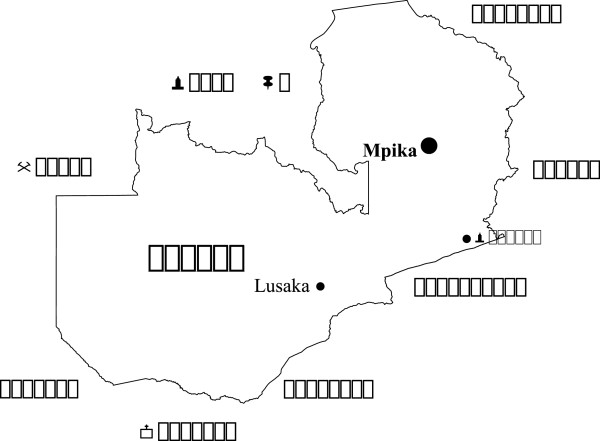
Map of Zambia showing the position of Mpika district.

**Figure 2:**
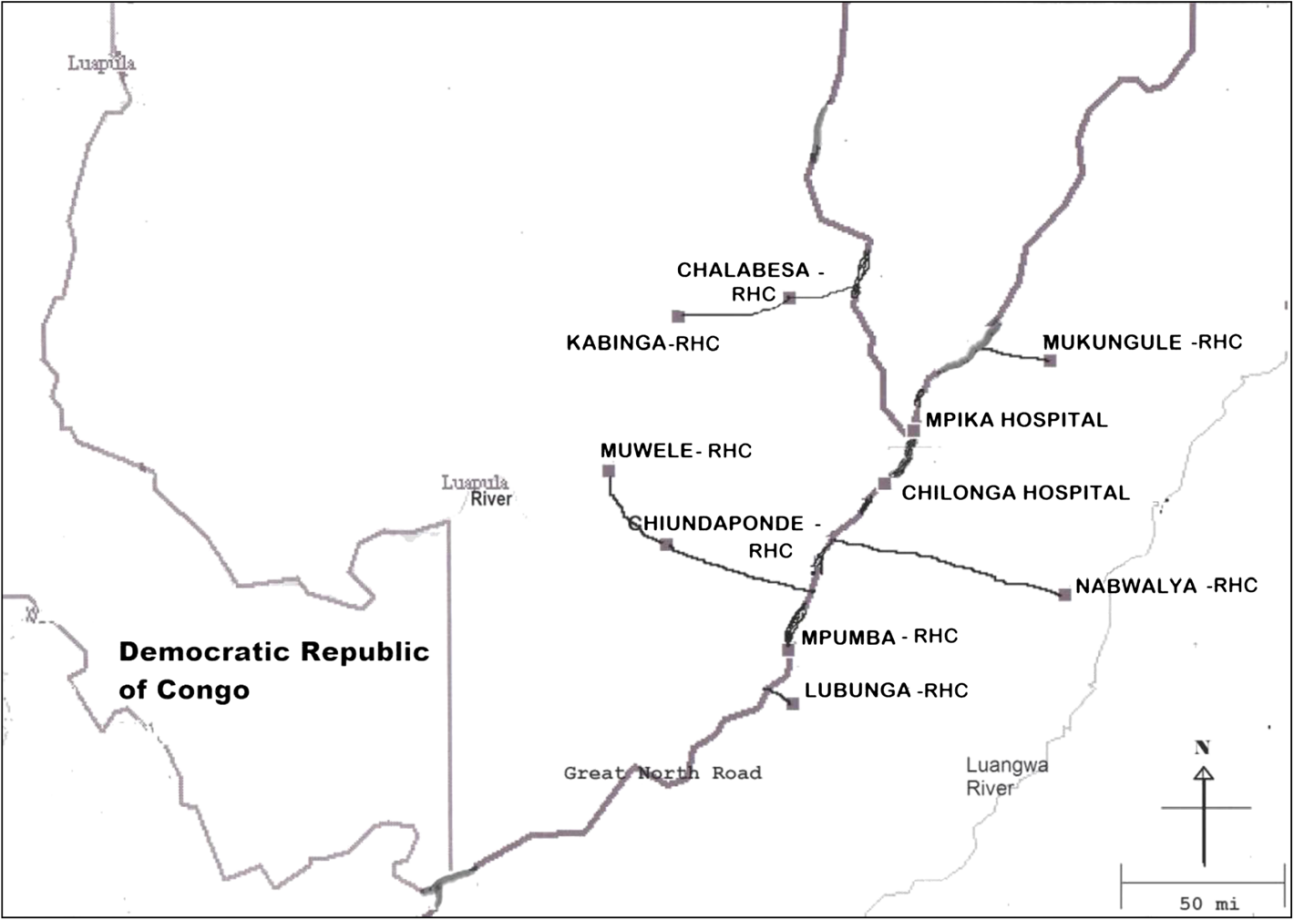
Location of the ten health institutions in Mpika district.

The position of Mpika district on the map of Zambia is shown in Figure 1. The location of the ten surveyed health institutions is shown in Figure 2. The coordinates and altitudes of the surveyed health institutions are in Table 1. Nabwalya RHC, with the least altitude, lies in Luangwa valley.

**Table 1:** Coordinates of the surveyed health institutions

**Health institution**	**Coordinates**	**Altitude (ft)**
1. Mpika District Hospital	S11 50.632 E31 26.722	4481
2. Chilonga Mission Hospital	S12 01.657 E31 20.394	4742
3. Nabwalya RHC	S12 25.117 E31 58.694	1895
4. Chiundaponde RHC	S12 14.596 E30 34.633	4123
5. Muwele RHC	S11 59.120 E30 22.600	3863
6. Mukungule RHC	S11 35.748 E31 55.378	3563
7. Chalabesa RHC	S11 22.562 E31 00.806	4195
8. Kabinga RHC	S11 26.587 E30 36.993	3910
9. Mpumba RHC	S12 36.969 E31 00.321	5056
10. Lubunga RHC	S12 48.027 E31 01.088	5001

### Questionnaire 1

This questionnaire, in form a written test, assessed the respondents on their general knowledge about HAT disease. A total of 28 health workers in the Outpatient Department (OPD) of the two hospitals and eight RHCs responded to the questionnaire/test on General knowledge on Human African Trypanosomiasis disease. The ranks and number of the respondents were as follows: Medical doctor (1), Medical Licentiates (3), Clinical Officers (6), Registered Midwife (1), Registered Nurses (2), Enrolled Midwives (3), Enrolled Nurses (8), and Environmental Health Technician (4). The respondents had to answer 14 questions. Out of the 28 respondents only 13 scored 50% and above, giving a pass rate of 46%. The mean score out of 14 was 6.7 with Standard Deviation of 3.6. The percentage of respondents with correct answers to the 14 questions were as follows: pathogen that causes HAT (42.9%), how HAT is transmitted (82.1), early symptoms and signs of HAT disease (57.1%), diseases with similar symptoms and signs as early HAT disease (67.9%), late symptoms and signs of late HAT disease (57.1%), diseases with similar symptoms and signs as late HAT disease (39.3%), stages of HAT disease (28.6%), how to make a diagnosis of HAT disease (64.3%), how to differentiate stages of HAT disease (0%), outcome of untreated cases of HAT disease (71.4%), drugs used to treat HAT cases (42.9%), route of administration of antitrypanosomal drugs (28.6%), side effects of antitrypanosomal drugs (39.3%), other methods of control of HAT apart from treatment (53.6%).

### Questionnaire 2

The responses to the questions in this questionnaire were as follows:

### Qualified health staff available to manage HAT cases per health institution

Table 2 shows the number of qualified health staff available to manage HAT cases at each surveyed health institution. Only Mpika District Hospital and Chilonga Mission hospital had medical doctors available to manage HAT cases.

**Table 2:** Qualified health staff available to manage HAT cases per health institution

**Institution**	**Medical doctor**	**Medical licentiate**	**Clinical officer**	**Registered nurse**	**Enrolled midwife**	**Enrolled nurse**	**Environmental health technician**
Chilonga Mission Hospital	3	1	6	-	-	4	-
Mpika District Hospital	1	2	1	-	-	4	-
Chiundaponde RHC	-	-	-	-	1	1	-
Muwele RHC	-	-	-	-	1	-	1
Mpumba RHC	-	-	-	1	-	1	1
Mukungule RHC	-	-	-	1	1	-	-
Chalabesa RHC	-	-	-	2	-	1	1
Kabinga RHC	-	-	-	-	-	1	-
Lubunga RHC	-	-	-	-	-	1	1
Nabwalya RHC	-	-	-	-	1	-	-

### Qualified health staff trained to manage HAT cases

Only staff at Chilonga Mission Hospital and Mpika District Hospital had been trained in management of HAT cases. At Chilonga Mission Hospital, three Medical Doctors, one Clinical Officer, and one Medical Licentiate had been trained locally. At Mpika District Hospital two Medical Licentiates and one Clinical Officer had been trained locally. None of the health staff manning RHCs surveyed had been trained in the management of HAT cases.

### Qualified health staff that have undergone refresher course on HAT management

The last refresher course organized by the World Health Organisation and Ministry of Health was in 2009. Only four medical staff from four health institution attended this course. These were one medical doctor from Chilonga Mission Hospital, one Medical Licentiate from Mpika District Hospital, one enrolled Midwife from Nabwalya RHC, and one Registered Nurse from Mukungule RHC.

### Availability of trypanosomicides in surveyed health institutions

Only Chilonga Mission Hospital stocked trypanosomicides in the whole district. It was reported that there were times when the institution ran out of trypanosomides. The only drug available at the time of the survey was suramin.

### Referral system

Chilonga Mission Hospital was the only institution that administered antitrypanosomal drugs to HAT patients in the district. The staff that administered the antitrypanosomal drugs was Medical doctors, Medical Licentiates, clinical officers, and trained nurses. Chilonga Mission Hospital was the final referral centre for all suspected HAT cases in the district even though some cases from RHCs such as Chalabesa were referred to Mpika District Hospital for laboratory confirmation of the cases. Even Mpika District Hospital referred confirmed HAT patients to Chilonga Mission Hospital for treatment since it does not stock antitrypanosomal drugs. No transport was provided for suspected HAT cases referred to either Chilonga Mission Hospital or Mpika District Hospital from RHCs.

### Laboratory facilities for HAT diagnosis

Only two health institutions in the district, Chilonga Mission Hospital and Mpika District Hospital, had functional laboratories that could diagnose HAT cases. Microhaematocrit centrifugation method (Woo’s method) and Giemsa Thick Smear Light Microscopy were used to diagnose HAT at Chilonga Mission Hospital while only Woo’s method was used at Mpika District Hospital. There was no External Quality Assurance program at either Chilonga Mission Hospital or Mpika District Hospital. Chalabesa RHC had a non-functional laboratory due to the non-availability of a trained laboratory technician/technologist.

### Qualified laboratory staff

Only Chilonga Mission Hospital and Mpika District Hospital had qualified laboratory staff. Chilonga Mission Hospital had two laboratory technologists and one laboratory technician. Mpika District Hospital had one Biomedical Scientist, two laboratory technologists, and one microscopist. Only one laboratory technologist from Chilonga Mission Hospital attended a refresher course on laboratory diagnosis of HAT which was organized by World Health Organisation and Ministry of Health in 2009.

### Discussion

This study has demonstrated the various challenges a lowly endemic country such as Zambia faces in implementing a successful elimination program for HAT.

The general knowledge on HAT disease of health staff was unsatisfactory for the proper early identification of clinical signs and symptoms suspicion of HAT and management of HAT cases in the surveyed health institutions. Clinicians in the forefront of managing HAT cases need to be abreast with improved standards in HAT patient care. In this case, refresher courses need to be organized by the Ministry of Health with the assistance of organizations such as the World Health Organization (WHO) every two years for medical doctors, nurses, medical licentiates, clinical officers, and Environmental Health Technicians in both hospitals and rural health centres in Mpika District and other districts at risk of HAT transmission in Zambia. It is absolutely necessary that there is awareness on HAT among health policymakers at the Ministry of Health so that they understand the importance of these trainings.

The questionnaire on Management of HAT cases revealed gross understaffing of essential health workers to clinically diagnose and adequately manage cases of HAT in the surveyed health institutions. Nabwalya RHC, which is the most HAT affected RHC only had one qualified enrolled Midwife who had to attend to all health problems of the surrounding community. It was also observed that the turnover of health staff in surveyed health institutions was high such that experienced qualified staff in HAT clinical diagnosis and management had moved on with inexperienced qualified staff taking their places. All the RHCs surveyed had no staff that had received specific training in HAT diagnosis and treatment. There is need for Ministry of Health to implement staff retention program to attract qualified staff to work in remote RHCs in Mpika District. There is need to motivate in kind health workers who are at the frontline in the early identification of clinical signs and symptoms suspicion of HAT at RHCs to encourage them to refer these cases to diagnostic and treatment centres [8]. This will compel them to be on the lookout for cases of HAT as they screen patients for other illnesses.

There was only one treatment centre for HAT cases in the district. There is need for the Ministry of Health to establish Mpika District Hospital as an additional treatment centre for HAT in the district. This would save the health staff at the RHCs and Mipika District Hospital itself from referring all the confirmed and suspected HAT cases to Chilonga Mission Hospital for confirmation of diagnosis and treatment. This would also decongest Chilonga Mission Hospital which attends to at least 200 patients in the outpatient department per day and thereby improve health care delivery at both hospitals. These two hospitals would also act as monitoring centres for HAT occurrences in the district since they both have laboratory facilities for the diagnosis of HAT.

In view of erratic supply of trypanosomicides at the only treatment centre for HAT in the district there is need for the Ministry of Health to ensure that drugs for both stages of the disease are in stock at all times. This will minimize deaths due to HAT which could be attributed to non-availability of effective trypanosomicides at the treatment centre. The WHO is always ready to assist countries endemic for HAT that need supply of trypanosomicides.

Only the two hospitals in the district had functional laboratories with qualified laboratory personnel. There is need for the Ministry of Health to capacitate RHCs that have non-functional laboratories through provision of laboratory equipment and qualified laboratory technicians to save suspected HAT patients from walking long distances to the two diagnostic centres in the district [8].

The commonly used diagnostic method for HAT diagnosis at the two hospitals was Giemsa Thick Smear Microscopy at both hospitals while Woo’s method was performed on request at one of the two Hospitals. Both these diagnostic methods are not sensitive enough to detect HAT cases [9]. In order to minimize missing cases of HAT it is essential that more sensitive diagnostic tests such as the improved Model of Mini Anion Exchange Centrifugation Technique (mAECT) are introduced at diagnostic centres [9]. mAECT should only be introduced at diagnostic centre once the laboratory technicians at these centres have been trained on how to use this technique.

Most of the laboratory personnel at the two hospitals had never attended refresher courses on HAT diagnosis. Laboratory personnel involved in diagnosis of HAT need to be updated on the laboratory techniques, current and new, in the diagnosis of this condition through refresher courses. Quality of results of laboratory diagnosis of HAT at the two hospitals was not guaranteed as both of them lack external quality assurance program. In order to ensure quality of laboratory results of HAT diagnosis there is need to have an External Quality Assurance program for the laboratories at the two hospitals [10,11].

None of the RHCs provided transport to ferry suspected HAT cases to the only treatment centre in the district for laboratory confirmation of the cases and treatment. This is in spite of the fact that all the RHCs were located very far from this treatment centre. With such a finding it may be assumed that a good number of referred suspected HAT cases are unable to cover the long distance to the treatment centre and therefore end up dying at home in their villages. A proper referral system needs to be in place to ensure that all suspected HAT cases referred by a RHC reach the designated diagnostic and treatment centre [12]. The District Health Office in Mpika needs to ensure that there is a vehicle available at all times to ferry suspected HAT cases, in addition to other health conditions such as maternity cases and others, from RHCs to the treatment centre.

In conclusion, Zambia, like all other HAT lowly endemic countries faces a number of challenges in eliminating this infection. Targeting humans for early identification of signs and symptoms of HAT, confirmed laboratory diagnosis and treatment of HAT cases will eventually lead to elimination of HAT through reduced transmission among humans, even though this could be insignificant in a short period of time. In addition efforts to control wild animal reservoirs and vectors (tsetse fly) are necessary for eventual elimination of HAT in endemic countries according to WHO suggestions. The challenges identified above, if addressed, might contribute to either elimination or near elimination of HAT in HAT lowly endemic districts in Zambia.

## Abbreviations

GPS: Global Positioning System; HAT: Human African Trypanosomiasis; LAMP: Loop-mediated isothermal amplification; OPD: Outpatient department; RHC: Rural health centre; WHO: World health organization.

## Competing interests

The authors declare that they have no competing interests.

## Authors’ contributions

VM conceptualized the study, collected the data, conducted analyses and wrote manuscript, PS helped in conseptualizing the study, supported the administrative conduct of the study, and reviewed manuscript, VC collected the data and reviewed the manuscript. All authors read and approved the final manuscript.

## Authors’ information

Victor Mwanakasale is currently a senior lecturer at Copperbelt University, School of Medicine, Ndola Zambia. He is the Corresponding author for this manuscript.

Peter Songolo is the National Program Officer-Disease control, at World Health Organisation, Country office, Lusaka, Zambia.

Victor Daka is a scientist at Tropical Diseases Research Centre, Ndola, Zambia.

## Supplementary Material

Additional file 1 **Questionnaire 1.** General knowledge on HAT diseases. Questionnaire 2: Management of HAT cases. Click here for file
