# Supplementary material for: Challenges in the control of Human African Trypanosomiasis in the Mpika district of Zambia
Source: BMC Res Notes. 2013 May 4;6:180. doi: 10.1186/1756-0500-6-180 (PMC3648427; doi:10.1186/1756-0500-6-180)
Supplement: Additional file 1 — Questionnaire 1. General knowledge on HAT diseases. Questionnaire 2: Management of HAT cases. [file 1756-0500-6-180-S1.doc]

**Availability of supporting data.**

The samples of the two questionnaires used in the survey are presented below:

**Questionnaire 1.**

**General knowledge on HAT diseases.**

Name of the Institution:

Date of the test:

Respondent’s serial number:

Rank of the respondent:

1. What causes Human African Trypanosomiasis (HAT) also called sleeping sickness?

2. How is HAT transmitted?

3. What are the early symptoms and signs of HAT diseases?

4. Which other diseases have similar symptoms and signs of early HAT disease

5. What are the late symptoms and signs of HAT disease?

6. Which other diseases have similar symptoms and signs of late HAT disease?

7. How many stages of HAT diseases are there?

8. How can you make a diagnosis of HAT disease?

9. How can you differentiate the stages of HAT disease?

10. What happens to a case of HAT if it’s not treated?

11. Name the drugs that are used to treat HAT cases?

12. How are these drugs given to HAT patients?

13. What are the side effects to these drugs?

14. What other methods are used to control HAT apart from treatment of cases?

**Questionnaires 2.**

**Management of HAT cases.**

Name of Institution:

Date of Interview:

Respondents’ names

Rank of the respondents:

**A. Trained clinicians.**

1. How many staff is available to manage HAT cases at this institution:

2. Categorise the staff if any:

3. Is this staff trained to manage HAT cases?

4. Has any of this staff undergone a refresher course on management of HAT cases?

5. If ‘Yes’ to Q.4. who was trained?

6. If ‘Yes’ to Q.4, when was the last refresher course held?

7. If ‘Yes’ to Q.4, who conducted this training?

8. If ‘Yes’ to Q.4, where was it held?

**B. Drug availability**.

9. Does your institution stock antitrypanosomal drugs?

10. If Yes to Q9, which ones?

11. Are there any antitrypanosomal drugs in stock at the moment?

12. If ‘Yes’ to Q.11, which ones?

13. What does your institution do when there is a stock out of antitrypanosomal drugs and there is a HAT patient requiring treatment

**C. Referral system.**

14. Is treatment of HAT cases instituted in this institution?

15. If ‘Yes’ to Q 14, who administers the antitrypanosomal drugs to HAT patients?

16. If ‘Yes’ to Q14, do HAT patients pay for this treatment?

17. If ‘No’ to Q14, where are the patients referred to for treatment?

18. If you have to refer HAT patients somewhere for treatment do you provide transport for them?

19. Are referred HAT patients given reference letters to take to where they are referred to?

20. If ‘Yes” to Q.19, who prepares the referral letter?

**D. Reporting system.**

21. Are all cases of HAT recorded in your records books and reported to the District Health Office?

22. If ‘Yes’ to Q.21, which HAT cases are recorded and reported (Suspected, laboratory confirmed, or both suspected and laboratory confirmed)?

**E. Diagnostic methods.**

23. Which method is used to diagnose HAT in this institution?

24. Do you have laboratory facilities for the diagnosis of HAT?

25. If ‘Yes’ to Q.24, which laboratory method is used to diagnose HAT?

26. If ‘Yes’ to Q.24, do you have problems in procuring laboratory consumables for HAT diagnosis

27. If ‘Yes’ to Q.24, which laboratory consumables frequently run out?

28. If ‘Yes’ to Q.24, who are the suppliers of your laboratory consumables?

29. If ‘Yes’ to Q.24, is funding to this institution adequate to enable you purchase laboratory consumables?

30. If ‘Yes’ to Q.24, when laboratory consumables are out of stock how do you make a diagnosis of HAT?

31. If ‘Yes’ to Q24 is the laboratory equipment adequate for HAT diagnosis?

32. If ‘Yes’ to Q.24, do you have a service contract for your laboratory equipment for HAT diagnosis?

33. If ‘No’ to Q.31, which equipment is lacking?

34. If ‘Yes’ to Q.24, is there an External Quality Assurance program for HAT diagnosis in your laboratory?

35. If ‘Yes‘ to Q.34, who provides this EQA program?

36. If ‘Yes’ to Q.35, who pays for this program?

37. If ‘Yes’ to Q.24, how many trained laboratory technicians/technologists do you have?

38. If ‘Yes’ to Q.24, have your laboratory technicians/technologists have ever attended any refresher courses on HAT diagnosis?

39. If ‘Yes’ to Q.38, when was the last refresher course held?

40. If ‘Yes’ to Q.38, who organized this workshop?

41. If ‘No’ to Q.24, how do you make a diagnosis of HAT?

**F. General enquiry.**

42. Do you have any suggestions on how to improve the detection and treatment of HAT cases in Mpika district?
